# Inspiring engineers

**DOI:** 10.1038/s44172-022-00013-8

**Published:** 2022-06-23

**Authors:** Alessandro Rizzo, Damien Querlioz, Liwen Sang, Wan-Ting Grace Chen, Carmine Galasso, Thanh Nho Do, Liangfei Tian

**Affiliations:** 1 Department of Electronics and Telecommunications, Politecnico di Torino, Turin, Italy; 2 Centre de Nanosciences et de Nanotechnologies, Université Paris-Saclay, Gif-Sur-Yvette, France; 3 International Center for Materials Nanoarchitectonics, National Institute for Materials Science (NIMS), Tsukuba, Japan; 4 Department of Plastics Engineering, University of Massachusetts Lowell, Lowell, MA USA; 5 Department of Civil, Environmental & Geomatic Engineering, University College London (UCL), London, UK; 6 Graduate School of Biomedical Engineering, UNSW, Sydney, NSW Australia; 7 Department of Biomedical Engineering, Zhejiang University, Hangzhou, China

## Abstract

On International Women in Engineering Day, members of our editorial board highlight individuals who have inspired them during their research careers.

In celebration of International Women in Engineering Day, here we present seven short tributes to researchers who have inspired members of our editorial board along their academic research path. Inspiration came from intellectual creativity, insight and passion for their research field, as well as mentorship and support during an early career stage.

## Naomi Leonard

**Figure:**
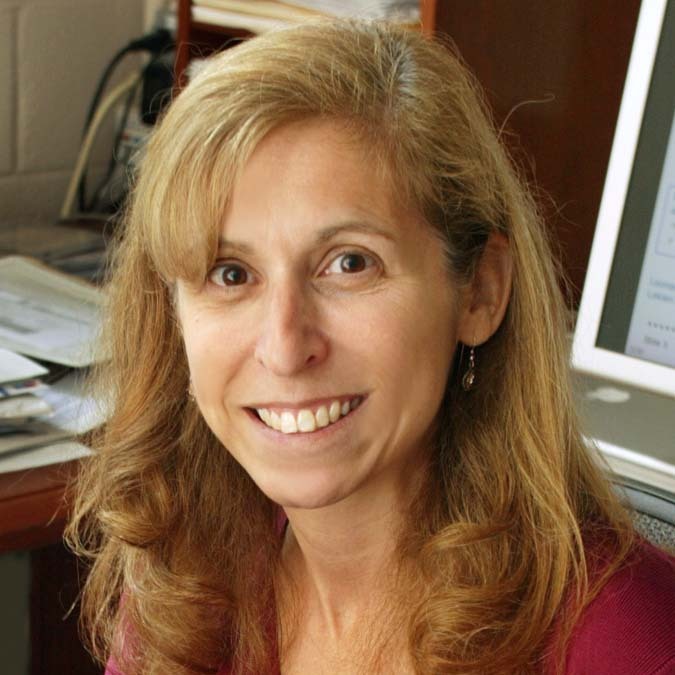
Frank Wojciechowski

Professor Naomi Ehrich Leonard works at the intersection of automatic control, nonlinear dynamics, complex systems and networks, and robotics. Her research focuses on the mathematical modeling and control of the decision-making and collective behavior of robots, animals, and humans. She bridges the gap between natural and engineered complex systems by developing analytically tractable mathematical models of collective dynamics. This allows for the exploration of the role of feedback control, communications, and heterogeneity in the collective behavior of networked units.

I met Professor Leonard when I attended a series of seminars she gave on the results of the Autonomous Ocean Sampling Network project, controlling ten underwater vehicles to form an automated and adaptive ocean observing system in Monterey Bay^1^.

Professor Leonard is the paradigmatic example of people who have succeeded in connecting distant universes, demonstrating that nature can inform engineering^2^ and, vice versa, engineering and mathematics can help in understanding nature^3^. A final word goes to her passion for dance and other performing arts—another opportunity for connecting nature and mathematics. In this regard, she is leveraging her knowledge in collective motion, experimenting with choreographers and composers using the “logic” of how groups move in nature and by design^4^. As a musician and a scientist, I am amazed by this activity and I am sure she will inspire me in pursuing my next endeavors.

Professor Leonard got her Ph.D. in Electrical Engineering from the University of Maryland. She is now the Edwin S. Wilsey Professor of Mechanical and Aerospace Engineering and an associate faculty member of the Program in Applied and Computational Mathematics at Princeton University. She is Director of Princeton’s Council on Science and Technology and an affiliated faculty member of the Princeton Neuroscience Institute and Program on Quantitative and Computational Biology. *By Alessandro Rizzo*.

## Elisa Vianello

**Figure:**
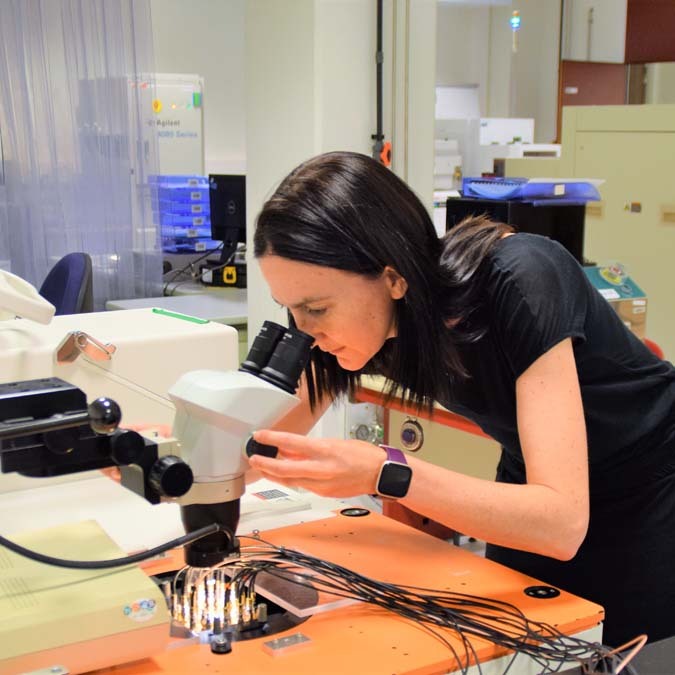
Utopik photo/CEA

Dr. Elisa Vianello is a senior scientist at CEA-Leti, an institute in France that acts as a bridge between the academic world and the microelectronics industry. She develops nanoelectronic technologies for bio-inspired neuromorphic computing with a special focus on resistive switching memory devices.

I have worked with Dr. Vianello for ten years. Her position is unique. She has the creativity of a leading-edge academic researcher. At the same time, she has a rigorous view from the microelectronic industry which allows her to truly understand if a technology will be viable in a system with millions of devices, not just in a proof-of-concept experiment. For this reason, her works have been most influential. She has introduced careful statistical evaluation methodologies in the field of neuromorphic computing and has designed artificial synapses, neurons, and content-addressable-memories that take full advantage of the properties of nanoelectronic devices. For these reasons, she has made me grow as a researcher. In our most successful joint work^5^, we turned the variability of nanomemories, their most major imperfection, into a desirable feature which allows a nanomemory-based system to learn to recognize cancerous images. Additionally, she is a wonderful mentor for her colleagues and her Ph.D. students, and simply a very good person.

Dr. Elisa Vianello received her Ph.D. degree in electrical engineering from the University of Udine in Italy and the University of Grenoble in France in 2010. She is an author on more than 100 technical articles and four book chapters. *By Damien Querlioz*.

## Tongjun Yu

**Figure:**
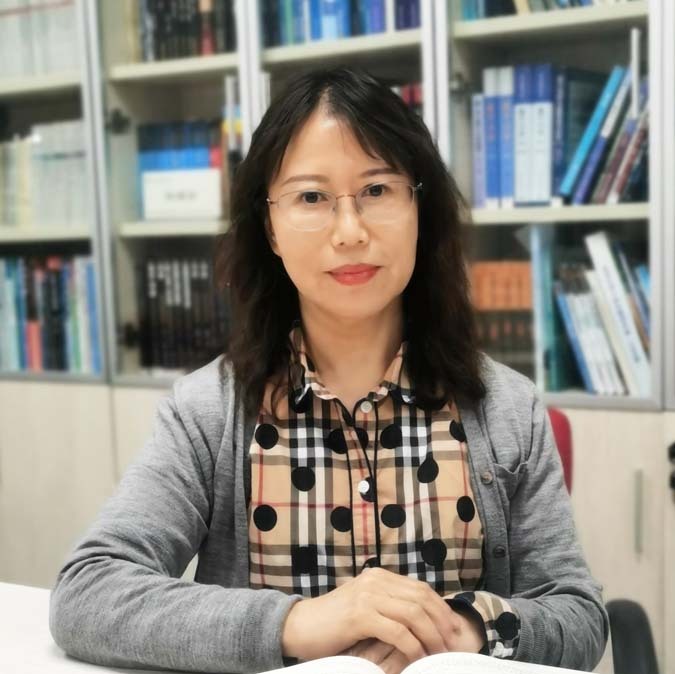
Tongjun Yu

Professor Tongjun Yu is a full professor at the School of Physics at Peking University, China where she develops III-V nitride semiconductor materials. She has championed material engineering in making deep UV light emitting diodes (LEDs), improving the optical properties of nitride-based devices. In her work, she identified a quantum-dot-like mechanism underlying the ultraviolet to blue color change in InGaN/GaN quantum-well LEDS, ultimately gaining control over their optical field^6^. She is also an expert in fabrication processes, having achieved the growth of low-defect-density, free-standing aluminum nitride crystals of up to 2 inches in size, thus advancing high-quality substrates for deep UV devices^7^. Her research has been influential in the commercialization of high-performance deep UV LEDs and solar-blind photodetectors, where their small size and high efficiency are used in sterilization, water purification, skin therapy, and other medical applications.

Professor Yu was one of my Ph.D. advisors at Peking University. Her passion for scientific research impressed and inspired me when I started my research career. During my studies, she inspired my research on interface engineering for deep UV LEDs and photodetectors. Later, she influenced my decision to become a researcher in Japan by sharing her own experiences and introducing me to Japanese research culture. Though I graduated long ago, we still meet when she visits Japan or attends conferences. Even now, she gives me advice on my career and research. She is always an advisor for me, not only for my work but also for my life.

Tongjun Yu obtained her Ph.D. at Tohoku University in Japan in 1999 and joined the faculty at Peking University in 2001. She has published more than 120 peer-reviewed papers and 30 patents, and has been awarded the Beijing Municipality Prize for Science and Technology. She has also achieved second place in the Science & Technology Development/Achievement Award from the Chinese Ministry of Education. *By Liwen Sang*.

## Jo Ann Ratto

**Figure:**
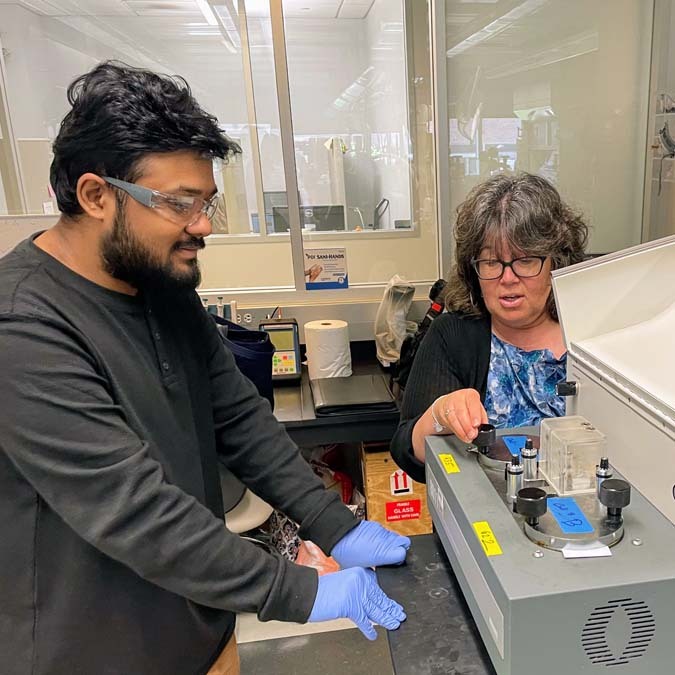
Jo Ann Ratto

Dr. Jo Ann Ratto has been a Materials Research Engineer at the U.S. Army Combat Capabilities Development Command Soldier Center (DEVCOM SC) for the past 33 years. Dr. Ratto’s research interests include biodegradable polymers and high barrier polymers for food packaging with emphasis on reducing solid waste for the military. In particular, Dr. Ratto’s research contributed to the design and improvement of the Meal Ready-to-Eat ration packaging, which have stringent requirements to assure performance in the field^8^. Her team developed melt-extrudable multilayer plastic films made from polymer structures which exhibit a high-barrier performance^9^. Her team was able to reduce the oxygen and water permeability by incorporating nanoparticle fillers, applying coatings, and/or fabricating oriented extruded films. Her contributions have enhanced our understanding of how to design biodegradable films for agricultural and food applications^10^.

Dr. Ratto has been a mentor of mine since I first joined the Department of Plastics Engineering at UMass Lowell in 2018. Since then, I have been fortunate to collaborate with Jo Ann on two projects, studying and developing packaging films. I was overwhelmed as an early-career faculty at that time, managing two projects in a new research field with multiple federal and industrial collaborators. Jo Ann taught me how to enter a new research field, showed me how to manage research projects between several parties, build successful collaborations, and work with students from different levels and backgrounds. She is a caring and trustworthy leader and mentor, helping mentees to achieve their career goals. In short, working with Dr. Ratto inspired me to be a better teacher-scholar.

Dr. Ratto obtained her B.A. in Chemistry from College of the Holy Cross, and master’s and doctorate degrees in Plastics Engineering from the University of Massachusetts Lowell. She has over 40 peer reviewed journal articles. Jo Ann retired in March 2022. *By Wan-Ting (Grace) Chen*.

## Fatemeh Jalayer

**Figure:**
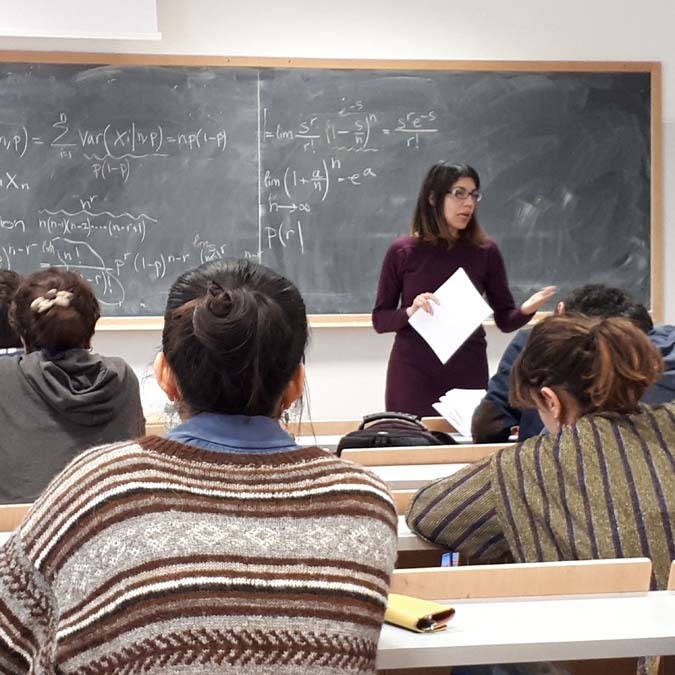
Fatemeh Jalayer

Professor Fatemeh Jalayer is an Associate Professor at the University of Naples Federico II. Her research focuses on probabilistic methods and computational tools for risk modeling and disaster risk reduction. She investigates the risks to buildings and infrastructure exposed to natural and anthropogenic hazards, including geohazards, rainfall-induced phenomena, fires and blasts. Her work enhances our collective ability to manage natural- and man-made-hazard risks.

Professor Jalayer and I first met when I started my Ph.D. in Earthquake Risk in Naples, and where she designed and delivered the first course on “Applied Statistics and Probability Analysis” in 2007. Here, she exposed many civil and structural engineering students to the field of probabilistic modeling and decision analysis. She immediately became and has since been an important role model for me. It is not a surprise that my current research, teaching portfolio and approaches align so well with some of the work and ideas I learned from Professor Jalayer. She also influenced my decision to move to and carry out my postdoctoral research in the USA by introducing me to some of the world-leading researchers in our field in California. Indeed, that move has been a very positive personal and professional life-changing event in my career. She is still a fantastic mentor for me and many other colleagues and young researchers and students. Professor Jalayer and I have continued collaborating over the past ten years.

Originally from Iran, Professor Jalayer pursued her Ph.D. at Stanford University. During this time, a paper she co-authored was awarded the 2003 Normal Medal of the American Society of Civil Engineers (ASCE)^11^. Since then, she has authored over 100 papers and has become a tenured professor in Italy. In addition to her research, she is the coordinator of the European Tsunami Risk Service (ETRiS) which is being integrated into the European Plate Observing System (EPOS)^12^. *By Carmine Galasso*.

## Allison Okamura

**Figure:**
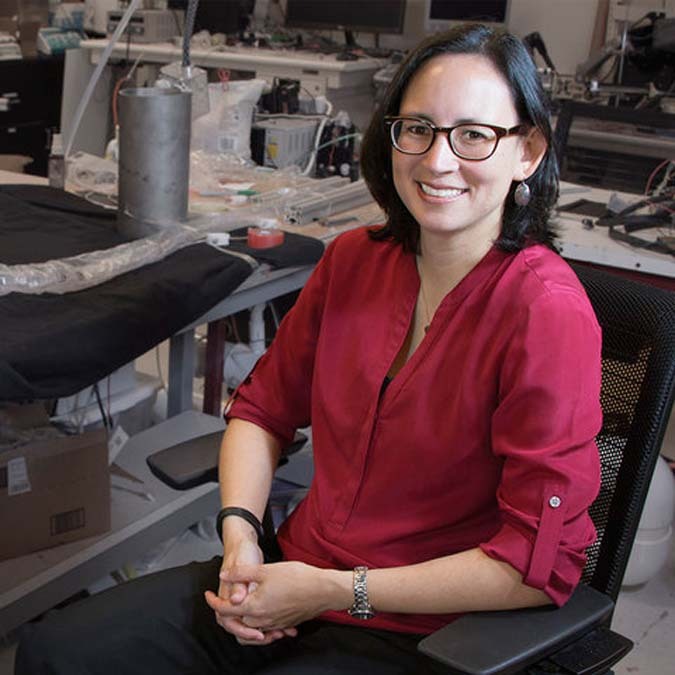
Standford Engineering/Amanda Law

Professor Okamura is a leader in wearable haptic devices, human-machine interaction and soft robotics. She is a pioneer in the understanding of how and to what extent information is transmitted via touch. She develops human-machine systems capable of haptic interactions for use in medical devices for surgery or prosthetics and applications in search and rescue robots. In soft robotics, she challenged systems of locomotion by developing a soft growing robot which elongates its body through the addition of new material, allowing for a tip-based length change on the order of more than thousands with navigational control^13,14^. Her use of biological principles has advanced engineering beyond what is possible in nature.

I met Professor Okamura several times at the IEEE International Conference on Robotics and Automation (ICRA) and Haptic Symposium conferences as well as during my postdoctoral fellowship at the California NanoSystems Institutes. Here, she explained how fundamental research in the human sense of touch has led to the development of novel haptic devices and soft robots. She kindly shared her reasoning behind targeting locations of the body other than fingertips. Particularly, she imparted the use of skin stretch devices^15^ which is now one of my current research directions. Outside of research, Professor Okamura actively participates in outreach providing people of all ages and backgrounds with the opportunity to discover the potential of robotics. Through this, she not only inspires me but also the next generation of robotics engineers.

Dr. Allison Okamura earned her Ph.D. at Stanford University where she is currently a professor. Her global research impact has been recognized by numerous awards, including the best paper awards at leading conferences such as ICRA and the Ten Robotics Technologies of the Year in 2018 by the journal Science Robotics. *By Thanh Nho Do*.

## Li-Zhu Wu

**Figure:**
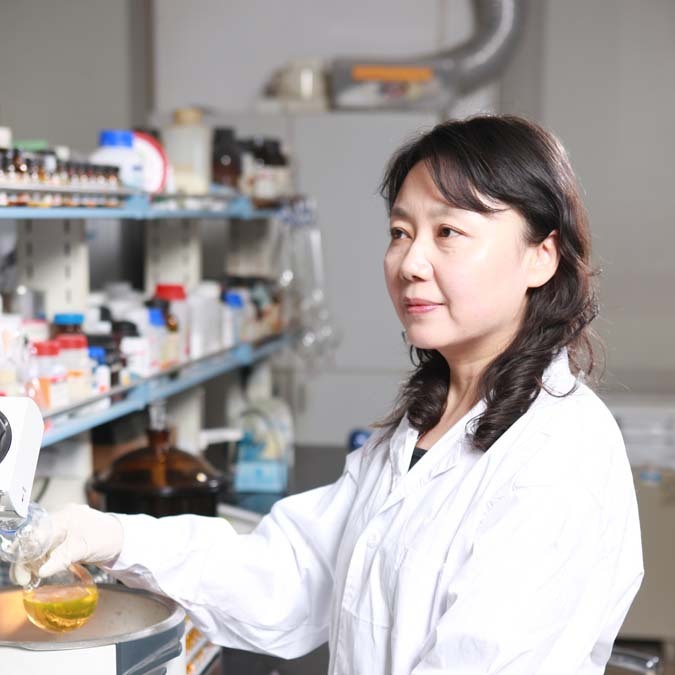
Li-Zhu Wu

Professor Li-Zhu Wu from the Technical Institute of Physics and Chemistry, China is a chemist and chemical engineer developing renewable energy technologies. She studies photochemical conversion, exploiting solar energy for the conversion of compounds to value-added products such as hydrogen, ammonia, and organic compounds. In one example of her work, she noticed that enzymes bind to substrates in elaborate pockets to increase the rate of a specific reaction under mild conditions. She introduced this idea to artificial hydrogen photogeneration. She reconstructed the confinement environment, surrounding the catalytic center and increasing the catalytic performance by a factor of 4000^16^. This work has influenced further research in photochemical synthesis and hydrogen production, key components of the future hydrogen economy.

I had the pleasure of attending a lecture by Professor Wu at Lanzhou University during my undergraduate studies. In her lecture, she introduced me to the concept of biomimetics for engineering artificial photosynthesis systems, including the now-famous research initiatives such as water splitting, carbon dioxide reduction and nitrogen fixation. She explained how to enhance the enantioselectivity of photochemical reactions using her novel catalytic systems. Her talk sparked my research interest in the field of biomimetics, which I ultimately chose for my Ph.D. studies and postdoctoral training. I still remember her advice that you should not give up easily when your research does not proceed well. I now share this with the students in my research group.

Li-Zhu Wu received her B.S. degree in chemistry from Lanzhou University and her Ph.D. degree from the Institute of Photographic Chemistry. She has been a member of the Chinese Academy of Sciences since 2019 and a Fellow of the World Academy of Sciences (TWAS) since 2022. *By Liangfei Tian*.
